# Advances and New Technologies towards Clinical Application of Oral Stem Cells and Their Secretome

**DOI:** 10.1155/2017/6367375

**Published:** 2017-01-24

**Authors:** Athina Bakopoulou, George T.-J. Huang, Mahmoud Rouabhia, Werner Geurtsen, Imad About

**Affiliations:** ^1^Department of Fixed Prosthesis and Implant Prosthodontics, School of Dentistry, Aristotle University of Thessaloniki, Thessaloniki, Greece; ^2^University of Tennessee Health Science Center, Memphis, TN, USA; ^3^Dental Faculty, University of Laval, Quebec, QC, Canada; ^4^Hannover Medical School, Hannover, Germany; ^5^Aix-Marseille University, CNRS, Institute of Movement Science (ISM), Marseille, France

Oral and dental diseases are major public health problems worldwide with profound effects on quality of life. Recent advances in tissue engineering have raised significant hopes for innovative alternative therapies based on regeneration strategies for tissues in the whole craniofacial area. The discovery and subsequent characterization of stem cells (SCs) from various oral sources, in conjunction with the cutting edge basic research of recent years towards understanding their biology, have expanded and deepened our knowledge of the complex role of SCs in developmental and repair processes. The application of this knowledge in translational studies has been a decisive milestone in bringing these technologies closer to clinical applications. Amazing technological advances, such as bioprinting for fabricating customized biomimetic scaffolds and tissue-specific organoids, development of bioreactors for manipulating the stem cell niche, use of microfluidics for single-cell analysis, and many others, have provided valuable tools for translating scientific advances into clinical settings. The establishment of Good Manufacturing Practice (GMP) protocols according to European regulations has facilitated feasibility and “proof-of-concept” clinical trials on the application of oral SCs in orofacial tissue regeneration. Most recently, the possibility of using the plethora of secreted trophic and immunomodulatory cytokines produced by SCs (secretome) as a therapeutic surrogate module for cells has been proposed as a safer alternative to stem cell transplantation, raising the therapeutic dilemma: stem cell or secretome therapy?

In this special issue, 9 papers focusing on advances and new technologies towards understanding the activation, recruitment, and clinical application of oral SCs and their secretome have been selected after being peer-reviewed from a number of submissions. These articles encompass multiple components of craniofacial tissue engineering, from teeth to salivary gland regeneration and neurovascular engineering.

Some of the papers present novel aspects of the biological characteristics of different oral stem cell populations and their potential to regenerate specific tissues. In particular, K. M. F. El-Sayed and C. E. Dörfer have coauthored a review article about gingival mesenchymal stem/progenitor cells (G-MSCs) as a promising tool to regenerate oral tissues. Using pertinent selections from the literature, the authors have provided a succinct overview of the isolation of G-MSCs, their characterization, and regenerative capacity. Although the available experimental data suggest potential therapeutic applications of G-MSCs, further studies are essential before the clinical use of these specific SCs could become a reality. Recent data also indicate that MSCs isolated from gingival tissue and dental follicles of children may have a multilineage differentiation potential, making them of increasing interest as an alternative source for oral SCs once the relevant gene expression pattern has been clarified. This led C.-M. Kang et al. to address this aspect in their study using 9 gingiva and 9 dental follicle biopsies from children. Their findings indicate that gingival tissue expressed more genes associated with keratinization, ectodermal development, and chemotaxis, while tissues from dental follicles showed a higher expression of genes involved in tooth and embryonic development. These results suggest that gingival tissue might be a good source for SCs to be used in regenerative dental procedures. In another innovative study, J. Xiong et al. investigated the cell surface proteome of human periodontal ligament stem cells (PDLSC) in comparison with other cell populations, such as human gingival fibroblasts and epithelial cells. In addition to the expression of well recognized MSC-associated cell surface antigens, such as CD73 and CD90, PDLSCs were also found to express two novel cell surface proteins: Annexin A2 and sphingosine kinase 1, which are not expressed in mature cells such as skin keratinocytes and play an important role in maintaining “stemness.” These proteomic findings provide a basis for further defining the cell surface protein expression profiles of PDLSCs. This, in turn, would enable further characterization of this cell population to facilitate novel isolation and purification strategies allowing their application in oral tissue engineering.

Other papers in this special edition focus on the neurogenic and/or neurovascular properties of dental stem cells, highlighting their importance in dental/orofacial tissue engineering. The neurogenic potential of dental pulp stem cells (DPSCs) has attracted intense interest as these cells spontaneously express neural markers in cultures. They have shown ability to differentiate into glial cells and functional neurons under specific conditions, even though different reports have been inconsistent indicating the need for further investigation and characterization of DPSC neurogenic potential. This special issue also included two articles investigating DPSCs for their neurogenic potential: one by F. I. Young et al. demonstrating for the first time the ability of single cell-derived clonal cultures of murine DPSCs (high nestin-expressing clone) to differentiate into immature neuron-like and oligodendrocyte-like cells in vitro. The membrane capacitance of the DPSC-derived neuron-like cells resembled that of cultured primary striatal neurons. The other article by J. Jung et al. demonstrated the use of xeno/serum-free condition to culture human DPSCs at early stage after DPSC isolation. The xeno/serum-free cultured DPSCs showed expression of PAX6, an important marker for cells committing to neural lineages. These cells also survived after transplantation into normal rat brain and injured spinal cord. The two articles indicate the importance of clonal selection and culture conditions on the neurogenic potential of DPSCs. In addition to these research articles, a review paper by J. Ratajczak et al. focuses on the neurovascular properties of dental stem cells (DPSCs, SHEDs, SCAPs, FSCs, and PDLSCs) and their importance in the vascularization and innervation that secures the viability of regenerated tissues in dental/orofacial tissue engineering. These authors provide a systematic analysis of the numerous extant studies on the ability of dental SCs to differentiate into endothelial cells and neural cell types, as well as on the angiogenic, neuroprotective, and neurotrophic effects of their secretome.

Innovative three-dimensional bioprinting nanotechnologies for SCs and their secretome have opened up previously unimaginable vistas for clinical application. J. N. Ferreira et al. have reviewed these exciting advances by focusing on salivary gland (SG) regeneration. This alleviates a wide range of medical conditions, from autoimmune to metabolic disorders, as well as damage after radiotherapy to treat specific head and neck cancers which lead to a poor quality of life due to xerostomia (dry mouth). These technologies are based on an ex vivo generation of organotypic cultures and SG organoids/miniglands using coculture systems to integrate the different SG cellular/tissue components, such as epithelial acinar and ductal cells, myoepithelial cells, and the networks of parasympathetic nerves, as well as ducts and vessels. This research improves our understanding of the properties of SC secretome and its potential use for SG regeneration.

Current research leading to the development of clinical-grade oral SCs and the application of GMP conditions, an essential step in the clinical application of dental SCs and their secretome, are concisely reviewed in another article coauthored by A. Bakopoulou and I. About. The salient biological properties of dental MSCs, critical for the translational pathway “from bench to clinic,” are presented. The capacity of these cells to reconstitute various dental and nondental tissues and the inherent osteo-/odontogenic, angiogenic, neurogenic, and immunomodulatory properties of their secretome are systematically described. Finally, key milestone achievements exemplifying their clinical utility in regenerative dentistry and initial data on well-designed clinical trials are discussed for the first time.

An original long-term clinical trial by S. Baba et al. provides novel data of phase I/II clinical trial on transplantation of autologous bone marrow SCs embedded into a 3D woven-fabric scaffold made of biodegradable poly-L-lactic acid resin fibers for treatment of chronic periodontitis. After SCs implantation into periodontal intrabony defects, a 36-month follow-up showed significant improvement of attachment level, pocket depth, and linear bone growth, providing solid clinical evidence for the application of SCs into the regeneration of oral tissues.

To conclude, this special issue highlights and encapsulates the amazing advances in knowledge and technologies regarding oral stem cell biology and tissue engineering. The aim has been to summarize the “state of the art” and consolidate the clinical utility of oral MSCs and their secretome in oral/craniofacial tissue regeneration. These innovative scientific data significantly improve our understanding of the potential risks involved in the use of these technologies, thus spurring efforts to surmount emerging problems and create a viable therapeutic option for the patient, the putative ultimate beneficiary of this groundbreaking technology.

